# Auditory Attention Activates Peripheral Visual Cortex

**DOI:** 10.1371/journal.pone.0004645

**Published:** 2009-02-27

**Authors:** Anthony D. Cate, Timothy J. Herron, E. William Yund, G. Christopher Stecker, Teemu Rinne, Xiaojian Kang, Christopher I. Petkov, Elizabeth A. Disbrow, David L. Woods

**Affiliations:** 1 Human Cognitive Neurophysiology Laboratory, Veterans Administration Northern California Health Care System, Martinez, California, United States of America; 2 Department of Neurology, University of California Davis, Sacramento, California, United States of America; 3 Center for Neurosciences, University of California Davis, Davis, California, United States of America; 4 Center for Mind and Brain, University of California Davis, Davis, California, United States of America; 5 Department of Speech and Hearing Sciences, University of Washington, Seattle, Washington, United States of America; 6 Department of Psychology, University of Helsinki, Helsinki, Finland; 7 Institute of Neuroscience, University of Newcastle, Newcastle upon Tyne, United Kingdom; 8 Department of Radiology, University of California San Francisco, San Francisco, California, United States of America; The University of Western Ontario, Canada

## Abstract

**Background:**

Recent neuroimaging studies have revealed that putatively unimodal regions of visual cortex can be activated during auditory tasks in sighted as well as in blind subjects. However, the task determinants and functional significance of auditory occipital activations (AOAs) remains unclear.

**Methodology/Principal Findings:**

We examined AOAs in an intermodal selective attention task to distinguish whether they were stimulus-bound or recruited by higher-level cognitive operations associated with auditory attention. Cortical surface mapping showed that auditory occipital activations were localized to retinotopic visual cortex subserving the far peripheral visual field. AOAs depended strictly on the sustained engagement of auditory attention and were enhanced in more difficult listening conditions. In contrast, unattended sounds produced no AOAs regardless of their intensity, spatial location, or frequency.

**Conclusions/Significance:**

Auditory attention, but not passive exposure to sounds, routinely activated peripheral regions of visual cortex when subjects attended to sound sources outside the visual field. Functional connections between auditory cortex and visual cortex subserving the peripheral visual field appear to underlie the generation of AOAs, which may reflect the priming of visual regions to process soon-to-appear objects associated with unseen sound sources.

## Introduction

The assumption that retinotopic visual cortex is activated exclusively by visual inputs has recently been challenged by brain imaging studies that have demonstrated auditory occipital activations (AOAs) in blind [1]–[8] as well as sighted subjects [9]. This study aims to answer two key questions regarding this phenomenon. First, given that AOAs are absent in most neuroimaging studies of audition, what specific aspects of auditory processing are critical for their occurrence? Second, what are the visual response properties of the occipital regions producing AOAs?

Evidence has emerged for direct anatomical connections between superior temporal and occipital regions that may play an important role in the crossmodal integration of sensory experience [10], [11]. These studies have revealed monosynaptic projections from core and parabelt fields of auditory cortex to V1 in the macaque, with the majority of connections terminating in regions that respond to visual stimuli in the peripheral field [10]. Similar connections have been reported in humans [12] and may help to explain the enhanced strength of sound-flash illusions in the visual periphery [13]–[15].

Evidence of AOAs was first reported in with congenitally blind individuals using event-related potentials [16]–[18]. Later, functional magnetic resonance imaging (fMRI) demonstrated AOAs in both early and late-blind subjects [2], [4], [8], [19]–[21]. Although AOAs have been occasionally reported in blind subjects performing non-spatial auditory discrimination tasks [21], [22], they are reliably found in blind subjects performing sound localization tasks [2], [6], [8], [20]. The presence of prominent AOAs in the blind may help to explain their superior performance on sound localization tasks [23]–[26]. Indeed, AOA magnitudes in blind individuals correlate with task performance in auditory localization [6] and non-spatial tasks [19]. In contrast to the prominent AOAs found in blind subjects, early studies typically found no AOAs in sighted subjects [4], [20] suggesting that AOAs may be a consequence of neuroplastic changes resulting from visual deprivation that enhanced auditory processing abilities of the blind [2], [16], [27]–[34]. However, a role for occipital visual cortex in spatial hearing in the normally sighted subjects has also been proposed on the basis of neuropsychological studies [35] as well as studies using TMS [36] and recent studies using fMRI [37].

AOAs have not been reported in the great majority of fMRI studies of auditory processing. Nevertheless, AOAs in normally sighted subjects have been incidentally reported in such diverse tasks as word perception [38], speech discrimination [39], sentence processing [40], detecting a subject's own name [41], intermodal selective attention [42]–[44], music discrimination [45], [46], attention to auditory components in auditory-visual speech [47], auditory sound discrimination [48], [49] and auditory spatial attention in the absence of visual stimuli [9]. While these tasks all require active listening to complex sound sources, it is unclear which cognitive or sensory aspects of auditory tasks are critical for the occurrence of AOAs. Do AOAs reflect the sensory analysis of particular sound characteristics in visual cortex, or do they reflect specialized cognitive operations associated with focused auditory attention?

The regions of visual cortex that generate AOAs also remain obscure. While fMRI studies have broadly localized AOAs to the cuneus [49]–[52] and lingual gyrus [46], [53]–[55] in Talairach coordinates, cortical surface mapping techniques are needed to localize AOAs to specific regions of visual cortex. In one recent study, Jack and colleagues examined task-related activations of visual cortex [56]. Cortical surface maps from individual subjects performing a tone-discrimination task showed widespread AOAs that were centered in peripheral regions of V1 (eccentricities greater than 6°). In the current study, we performed population-based cortical surface mapping to localize AOAs to precise areas of visual cortex with known response properties, in order to elucidate the functional role that AOAs might play during active listening.

A primary focus of the current study was to compare the role of acoustic and cognitive factors in AOA generation. To this end we applied an intermodal selective attention paradigm originally designed to elucidate the functional properties of auditory cortex [57], [58]. To characterize acoustic effects, sounds varied in frequency, location, and intensity in different stimulus blocks. Subjects performed demanding auditory or visual tasks with either unimodal or bimodal stimulus sequences, which were then contrasted to characterize the effects of attention. To ensure that AOAs were not dependent on the idiosyncratic characteristics of the tasks, we used a wide range of stimuli, including different tone patterns and two kinds of visual stimuli (faces and words).

Reliable AOAs were found in regions of visual cortex subserving the far visual periphery. We analyzed the relationship between AOAs and performance on auditory tasks and also performed event-related analyses to evaluate the possible relationship between AOAs and task-related cognitive operations such as target detection and task switching [56]. In addition, we used functional connectivity analyses to investigate the relationship between AOAs and modality-specific attentional modulations occurring in visual and auditory cortex. The results suggest that the activation of peripheral visual cortex is an essential component of a cortical network subserving sustained auditory attention.

## Methods

### Ethics statement

All subjects provided informed consent in accordance with the VANCHCS Institutional Review Board.

### Subjects

Nine individuals (aged 18–34 years, 8 male, 2 left-handed) each participated in one orientation session that included task training and anatomical imaging and then underwent six separate 1-hr fMRI sessions (three with sparse and three with continuous sampling) over a period of 2–6 weeks. All subjects had normal or corrected-to-normal vision and normal hearing.

### Stimuli

**Figure 1:**
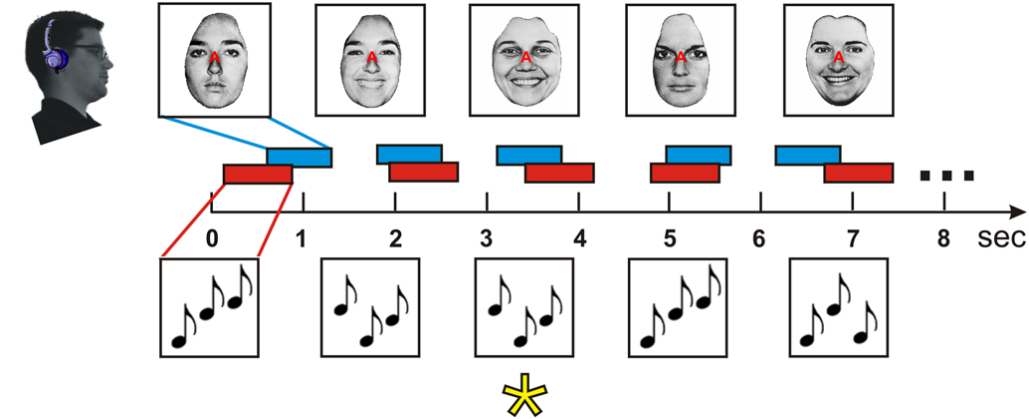
Stimuli and task. Subjects attended to either auditory or visual stimuli in 21 s blocks to detect repeated stimulus events in the modality cued by a letter at fixation (top row). Auditory and visual stimuli occurred asynchronously at mean stimulus onset intervals of 1.5 s within each modality. Auditory targets (asterisk) were repeated tone triplets (250 ms/tone = 750 ms, red rectangles). Visual stimuli were presented for 700 ms (blue rectangles).

Functional images were acquired while subjects performed attention-demanding one-back matching tasks in the attended modality (Figure 1) cued by a partially transparent cue letter (“A” or “V”) at fixation indicating the modality to be attended. Stimuli were presented in blocks that used unimodal or bimodal stimulation. In unimodal auditory and visual blocks (UA and UV, respectively), subjects always attended to the presented modality. In bimodal blocks, auditory and visual stimuli were presented concurrently, and subjects were cued to attend to the auditory (BA blocks) or visual (BV) modality. During bimodal sequences auditory and visual stimuli were presented asynchronously with randomized temporal relationships to minimize intermodal integration. The four types of blocks (UA, UV, BA, BV) occurred with equal frequency.

Auditory stimuli were tone triplets of 750 ms duration generated by selecting pseudorandomly and exhaustively from three 250 ms tones. Target stimuli were triplet repetitions occurring with a probability of 0.1. The tones were separated by 3-semitone steps and centered on frequencies of 225, 900, or 3600 Hz in different blocks. In each block, tones were delivered at either 70 or 90 dB SPL, and to either the left ear, right ear, or both ears according to a randomized design. Tones were presented over continuous broadband 70 dB SPL masking noise through insert earphones. Ambient scanner noise was further attenuated with circumaural ear protectors. Visual stimuli in each block were black and white photographs of faces (visual angle 2°×3°) or words (mean visual angle 2.5°×0.8°). Faces were eight individuals from the Ekman set [59], each with four different facial expressions (disgust, fear, happiness, and neutral). Targets in the face blocks were successive photographs of the same individual with different emotional expressions. Words were selected from ten different semantic categories (e.g., cities, plants, animals, etc.), each with four exemplars. Targets in the word blocks were successive words belonging to the same semantic category. Responses were recorded to measure reaction times (RTs) and to permit the calculation of hit and false alarm rates. Stimulus presentation and response collection were controlled with Presentation software (NBS, Albany, CA.).

Retinotopic mapping of the visual cortex was performed with two subjects. The horizontal and vertical meridians were mapped using high-contrast checkerboard wedges (extending from 0.2° to 4.79°, 0.05° wide at inner edge, 0.58° wide at outer edge), and two eccentricities were mapped using central (0.96° eccentricity, 0.19° wide) and peripheral (4.79°, 0.38° wide) rings.

### MRI Scanning

High-resolution T1 anatomical images were acquired from each subject on a 1.5 T Philips Eclipse scanner (matrix size 256×212×256, voxel size 0.94×1.30×0.94 mm, TE 4.47 ms, TR 15 ms, flip angle 35°, field of view 240×240 mm). Six separate functional imaging sessions were performed with each subject using an EPI sequence (matrix size 128×128×29, 29 axial slices 4 mm thick plus 1 mm gap, voxel size 1.88×1.88×5 mm, TE 39.6 ms, flip angle 90°, FOV 240×240 mm). All functional scans used a similar blocked design (16 behavioral trials/block). In three sessions for each subject images were acquired using a sparse imaging sequence (2 functional images acquired per block, TR 10.4 s, 20.8 s/block, sequential slices) to reduce acoustic noise [60]. The other three sessions employed continuous imaging (8 functional images per block, TR 2.9 s, 23.2 s/block, interleaved slices) to permit the analysis of the time course of activations. Functional data sets from sparse and continuous imaging were analyzed separately for each subject.

**Figure 2:**
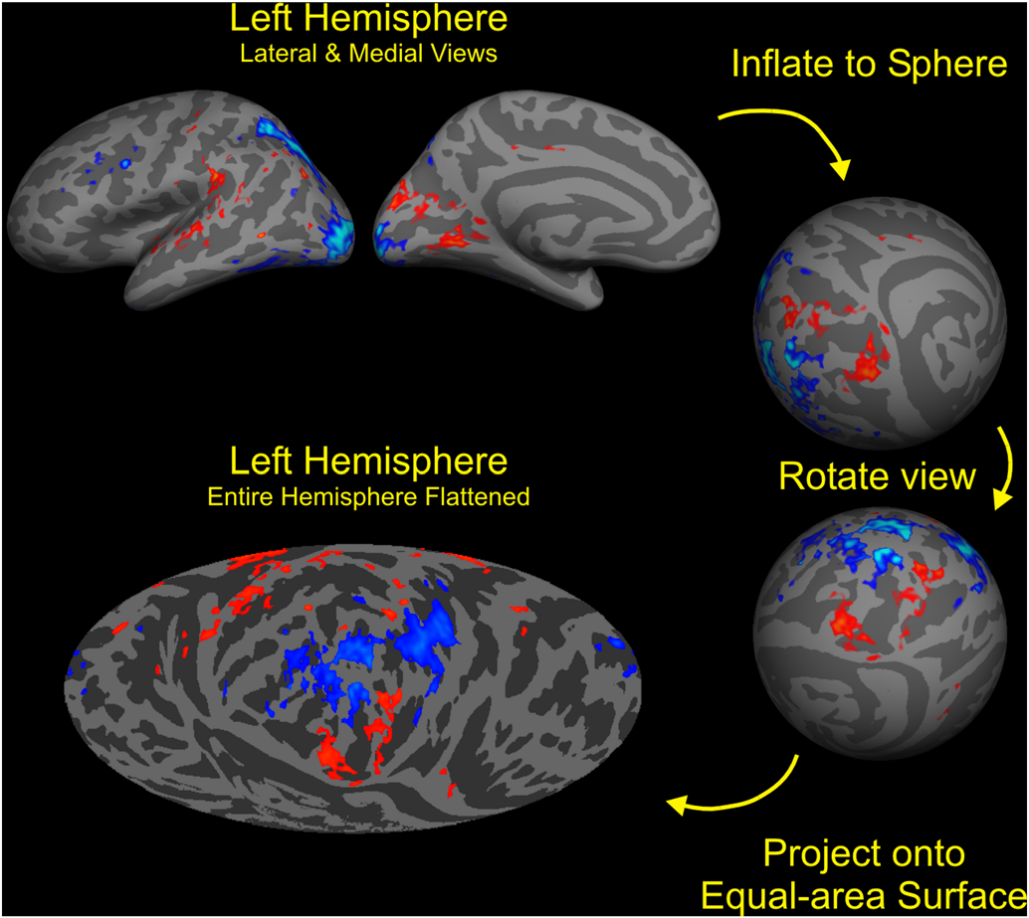
Cortical surface analysis display method. Schematic diagram showing the transformation of a cortical hemisphere partially inflated using FreeSurfer to the equal-area Mollweide projection flat map used to display the data in this study. Clockwise from top left: Views of the medial and lateral surface of a semi-inflated model of the cortical surface (gray matter/white matter boundary) of the left hemisphere averaged over 60 individual brains. Shading indicates average cortical curvature (light: convex; dark: concave) with an overlaid functional activation map showing the effects of attention (see Figure 4 for more details). Next, the hemisphere is fully inflated to a sphere using FreeSurfer, and rotated to place the posterior occipital lobe at the equator. Finally, the surface of the sphere is visualized using an equal-area Mollweide projection, with the occipital pole at the map's center.

We used cortical surface mapping procedures to analyze the AOA distributions in relation to cortical gyral and sulcal anatomy (Figure 2). Anatomical image sets were resliced to 1 mm^3^, segmented, inflated and coregistered to a spherical coordinate system using FreeSurfer [61]. Each subject's functional images were coregistered and resampled directly into the high-resolution anatomical space [62] after correcting for head movement using SPM5 [63]. Functional image data were high-pass filtered with a cutoff of 0.005 Hz using polynomial detrending. Activations in voxels corresponding to the cortical surface were quantified in native 3D space and visualized on the spherical surface using an equal-area Mollweide projection. Functional activations were superimposed on maps of the mean surface curvature of 60 healthy control subjects' whole-head T1 scans and displayed on equal-area Mollweide 2D projections of the spherical mean surface curvature maps.

### Behavioral Data Analysis

Subjects performed a difficult one-back matching task in the auditory or visual modality. Repeated-measures ANOVAs were performed to examine the differences between auditory and visual task performance. Data from auditory and visual tasks were grouped together to form a “modality” factor, which was crossed with imaging protocol (sparse or continuous) in a factorial design. The effects of intermodal attention were analyzed using the two bimodal conditions (BA and BV). Because the stimuli presented in these conditions were identical, every independent factor was included in this analysis: modality of attention; imaging protocol; auditory stimulus intensity, ear of delivery and frequency; and visual stimulus type.

### fMRI Data Analysis

#### Preprocessing

Percent signal change was calculated relative to the overall mean BOLD response for each voxel. Mean BOLD responses associated with each block were calculated by averaging across both functional images from the sparse imaging sessions and across images 2–8 (i.e., beginning 5.8 s after beginning of block) in continuous imaging sessions. Spatial smoothing was applied to the cortical surface data using a 3-mm FWHM Gaussian filter [64].

#### Stimulus-Dependent Activations (SDAs) and Attention-Related Modulations (ARMs)

**Figure 3:**
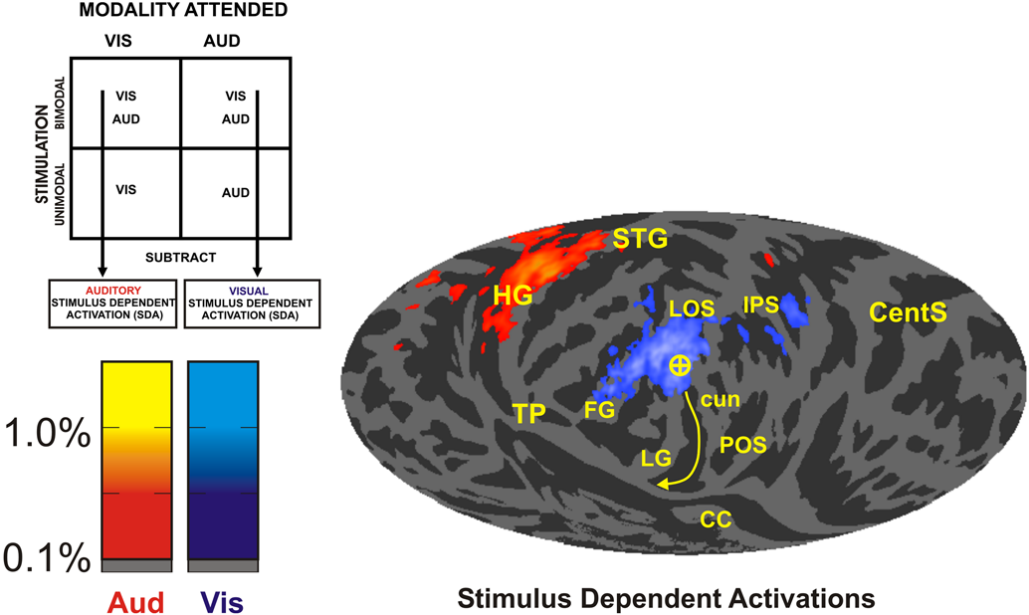
Stimulus-dependent activations. Stimulus-dependent activations (SDAs) to unattended stimuli projected on a map of mean curvature across both hemispheres (darker gray = sulcus). A circled cross indicates the occipital pole. The calcarine sulcus is indicated by the yellow arrow pointing away from the foveal towards the peripheral visual field regions. HG Heschl's gyrus, STG superior temporal gyrus, IPS intraparietal sulcus, CentS central sulcus, TP temporal pole, FG fusiform gyrus, LG lingual gyrus, cun cuneus, POS parietal-occipital sulcus, CC corpus callosum. Data from sessions using sparse image acquisition. All activation maps are triple-thresholded (z>3/p<0.001, signal change >0.1%, cluster size >20 voxels).

**Figure 4:**
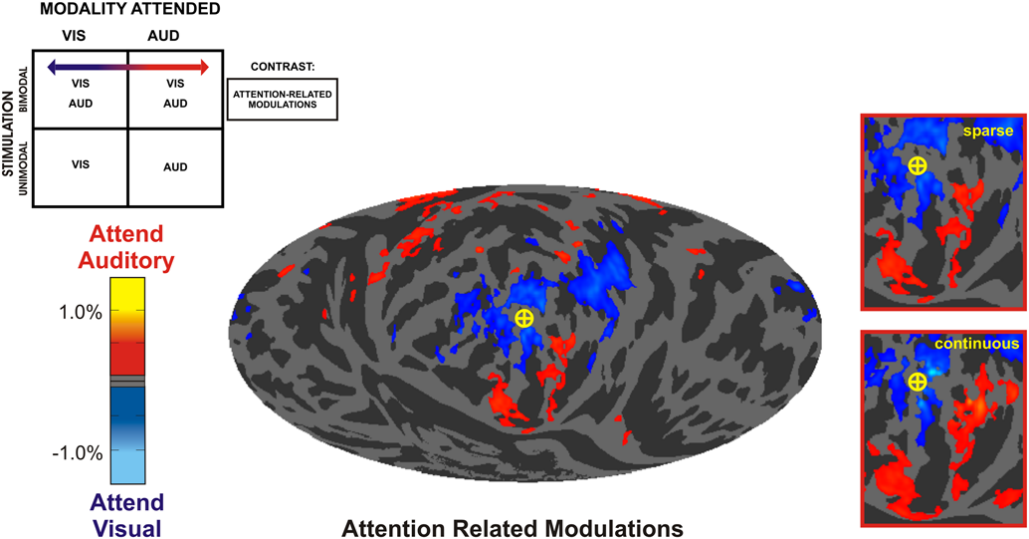
Attention-related modulations. Visual attention-related modulations (ARMs, blue) were seen in posterior occipitotemporal areas and the IPS. Auditory ARMs (red) were found in auditory cortex along the superior temporal plane with additional foci in the lingual gyrus and cuneus (auditory occipital activations: AOAs). The color scale shows mean percent signal change. Insets (right): mean occipital activations from sparse and continuous image acquisition sessions.

Statistical contrasts were used to identify stimulus-dependent activations (SDAs; activations related to unattended stimuli; see Figure 3) and attention-related modulations (ARMs; see Figure 4). SDAs were obtained by subtracting activations in unimodal conditions from activations in bimodal conditions that differed from the unimodal conditions only by the addition of task-irrelevant stimulation in the unattended modality. Hence visual SDAs were obtained by subtracting signals in UA blocks from signal in BA blocks, while auditory SDAs were obtained by subtracting signals in UV blocks from those in BV blocks. ARMs were identified by contrasting BV and BA blocks. These contained identical stimuli, and differed only in the modality attended.

#### Retinotopic Mapping

**Figure 5:**
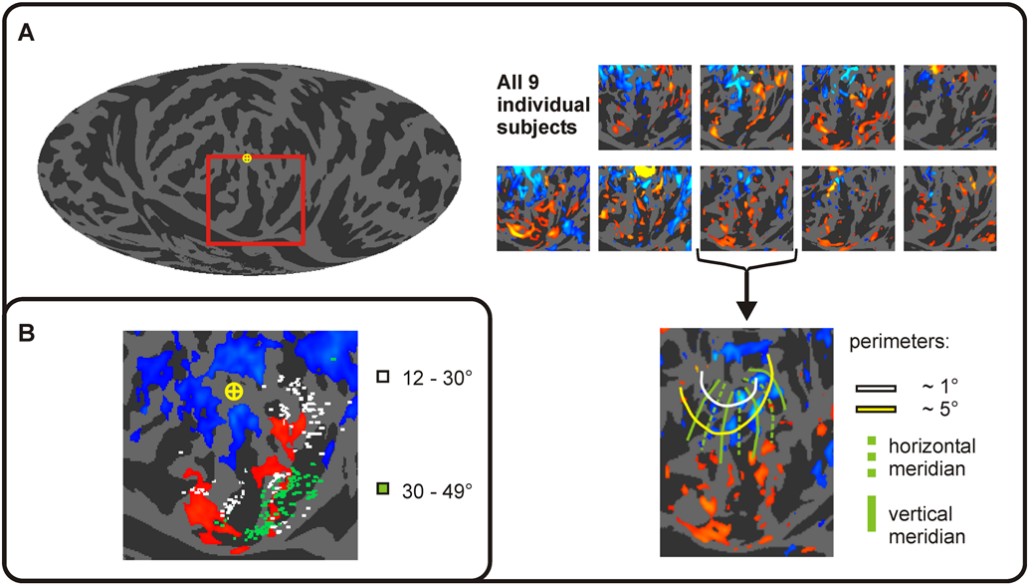
Occipital regions activated by auditory attention. (A) Left: average cortical surface anatomy showing occipital regions (box). AOAs in all 9 subjects, depicted on maps of their individual occipital cortex surface curvature. Bottom right: the activation map from one subject who underwent retinotopic mapping of the horizontal and vertical meridians (green lines) and two eccentric annuli (white and yellow lines). (B) Cortical surface projections of the Talairach coordinates reported by Stenbacka et al. (2007) for visual checkerboard patterns presented at 12–30° and 30–49° in the peripheral visual field, superimposed on the mean AOA map averaged across subjects. Dots represent the reported Talairach coordinates (white, 12–30°, green, 30–49°) projected to the closest corresponding location on the cortical surface for each of 60 brains in the anatomical database.

To compare the regions of visual cortex showing AOAs with the retinotopic representation of the fovea we mapped the vertical and horizontal meridians and retinal eccentricities up to 5° in two subjects using counterphase flickering (8 Hz) checkerboard patterns [65]. Since AOAs appeared to fall beyond the maximal eccentricity that could be mapped (5°), we additionally compared AOA distributions with those of activations produced by visual stimuli in the far peripheral field (up to 49° eccentricity) reported by Stenbacka and Vanni [66]. Due to the variable relationship between gyral structure and stereotaxic coordinates in individual subjects [67] we projected the Talairach coordinates from Stenbacka and Vanni to the nearest point on the cortical surface for each individual in the control database of 60 whole-brain T1 scans (white and green dots in Figure 5). We also measured the 3D Talairach coordinates of AOA maxima in the cuneus and lingual gyrus for both hemispheres.

#### Region of Interest (ROI) Analysis

We used a region of interest (ROI) analysis to evaluate the reliability of AOA generation and to test whether the AOAs were implicated in perceptual analysis of sensory information or in attention-related cognitive processes. ROIs were defined using the data acquired during sparse fMRI acquisition and their responses were analyzed using the independent data set obtained in sessions using continuous imaging. ROI voxels were required to meet three criteria: percent signal change from baseline (0.1%), statistical significance of the ARM contrast (z>2.97, p<0.001, uncorrected, in a fixed-effects analysis) and minimum cluster size (20 contiguous surface voxels). The last two criteria combine to control hemisphere-wide error at p<0.05 (fixed effects analysis) [68]. Two ROIs in pericalcarine visual cortex were chosen for analysis: (1) an AOA region, including the clusters in the lingual gyrus and cuneus, and (2) a central vision region in the posterior calcarine sulcus based on the visual ARM cluster in this area.

Three distinct repeated-measures ANOVAs (treating subjects as a random factor) were performed to test the significance of the ARM and SDA effects using the continuous imaging data. The effects of intermodal attention (i.e. the ARMs) were verified in an ANOVA using the data from the two bimodal conditions (BA and BV). Separate analyses were also performed using data from either the auditory (BA and UA conditions) or visual (BV and UV) attention conditions' data alone, in order to compare activations in the presence and absence of stimuli in the unattended modality.

#### Task-switching Activation Analysis

We evaluated the hypothesis that AOAs might reflect cognitive operations associated with task switching at block boundaries [56] by analyzing event-related time course regressors modeling the beginning and end of bimodal stimulus blocks where attention switched from the auditory to the visual modality or vice versa. Event-related time course regressors were created to model the BOLD response produced when subjects switched between performing the auditory and visual tasks. Task-switching events were modeled as square waves beginning at the conclusion of one block and ending 2 seconds later in the following block. Switching events were included for the transitions between all temporally adjacent bimodal blocks with different task modalities. These boxcar time courses were convolved with a standard, bigamma hemodynamic response function [69]. A fixed-effects t-test assessed the fit between the modeled and observed BOLD time courses for each surface voxel. T-maps were double-thresholded using statistical significance (t>3) and cluster size (20 contiguous surface voxels) as criteria.

#### Response-related Activation Analysis

Event-related time course regressors were also used to determine whether AOAs primarily reflected detection of the unpredictable auditory targets. The measured time course of subjects' button press responses associated with auditory target hits were convolved with a hemodynamic response function (HRF) for both the sparse and continuous imaging sessions. These target-related regressors were contrasted with regressors representing the periods during which subjects made no responses. Within auditory attention blocks, response events were modeled as positive square waves spanning the 750 ms prior to a recorded response, and non-response epochs (of variable length, spanning the intervals between each two response events) were modeled as negative square waves. The resulting two boxcar time courses were normalized to have equal energy, summed together, and were convolved with the standard HRF. A fixed-effects t-test assessed where the time courses for each surface voxel was non-zero. T-maps were double-thresholded using statistical significance (t>3) and cluster size (20 contiguous surface voxels) as criteria.

#### Functional Connectivity Analysis

The results from the analyses described below revealed that AOAs were positively correlated with sustained auditory attention and negatively correlated with activations in central visual areas during auditory attention conditions. However, because subjects switched attention between auditory and visual stimulus blocks, there was no truly activation-independent baseline. Thus, it is possible that AOAs could reflect relative deactivations of peripheral visual regions due to foveal attention during visual attention blocks [70], [71] rather than activations of peripheral visual regions during auditory attention blocks. If AOAs reflected the absence of inhibition during auditory blocks, one would predict a significant negative correlation between BOLD signal in the posterior (foveal) visual cortex and the AOA ROI. Alternatively, if AOAs were part of a cortical network activated during auditory attention, AOAs should be unrelated to activity in central visual field regions of visual cortex but correlated with activations in auditory cortex. We therefore also tested the hypothesis that there was a positive correlation between responses in the AOA ROI and auditory cortex.

We computed partial correlations [72] of the AOA ROI time series with time series of both the entire cortical surface and other ROIs [73]. In order to find consistent correlation values across subjects (i.e. a random effects analysis) we computed partial correlations for each subject separately, converted those to normally-distributed z-scores using the standard Pearson product moment distribution, and then performed a t-test that indicated whether mean z-score was significantly different from zero. We first computed the partial correlations of the AOA ROI with every voxel on the cortical surface during unimodal visual blocks while partialling out the global fMRI signal (the mean of the entire cortical surface) and the three main head motion correction components. Second, we calculated the partial correlation under all task conditions between the AOA ROI and an auditory cortex ROI in the same hemisphere defined from sparse data (see Supplemental Figure S1) while partialling out (1) the global signal and head motion parameters, (2) an ROI from both hemispheres defined as all visual ARM voxels in the posterior occipital region, and (3) indicator variables for bimodal vs. unimodal blocks and for auditory vs. visual blocks. The first cortical surface partial correlation examined whether there were significant correlations between the AOAs and the posterior occipital region, while the latter ROI-based partial correlation was designed to test the hypothesis that there were correlations between the AOAs and auditory cortex that could not be explained by visual functional activations or by any of the attention block conditions.

## Results

### Behavioral tasks

Hit rates were similar in auditory and visual blocks (62% vs. 67%, F_(1,8)_ = 2.67, p>0.10). During auditory conditions, subjects were more accurate in blocks with high- than low-intensity sounds (F_(1,8)_ = 16.09, p<0.005). The auditory hit rate was not significantly affected by the presence of visual distractors (F_(1,8)_ = 0.10).

### Activations to unattended auditory and visual stimuli

Figure 3 shows SDAs on the average inflated cortical surface. Visual SDAs (blue, cyan) were localized to the foveal region of retinotopic cortex and surrounding parafoveal zones with additional activations seen in higher visual areas in the temporal and occipital lobes and the intraparietal sulcus. Auditory SDAs were restricted to auditory sensory cortex on Heschl's gyrus and in surrounding regions on the superior temporal plane. There was no evidence of auditory SDAs in occipital cortex.

### Attention-related modulations

Figure 4 shows attention-related modulations (ARMs), isolated by contrasting activations from bimodal visual attention blocks with activations from bimodal auditory attention blocks. Areas showing enhanced activations during visual attention (blue/cyan) included the retinotopic areas in central calcarine cortex as well as higher visual areas in the lateral occipital sulcus, the fusiform gyrus, and the intraparietal sulcus.

Auditory ARMs were predictably prominent in auditory association cortex along the superior temporal gyrus (STG). In addition, auditory ARMs were evident in the cuneus and lingual gyrus (red/yellow, Figure 4). These AOAs occurred in peripheral visual cortex anterior to the regions that showed visual ARMs. AOAs had similar amplitudes and distributions in fMRI sessions using continuous and sparse image acquisition (Figure 4, insert) and were observed in every subject (Figure 5).

### Occipital regions generating AOAs

The results from one subject's retinotopic mapping are shown in Figure 5. AOAs in both subjects occurred in regions that were more peripheral than the maximal 5° eccentricities. AOA peaks occurred at Talairach coordinates of x = −6, y = −88 and z = 16 in the cuneus (lower visual field) and x = −10, y = −56 and z = −3 in the lingual gyrus (upper visual field). AOA foci corresponded to activations in the far peripheral regions of retinotopic cortex between the eccentricities of 12° and 49° as mapped by Stenbacka and Vanni [66].

### Region of Interest Analysis

**Figure 6:**
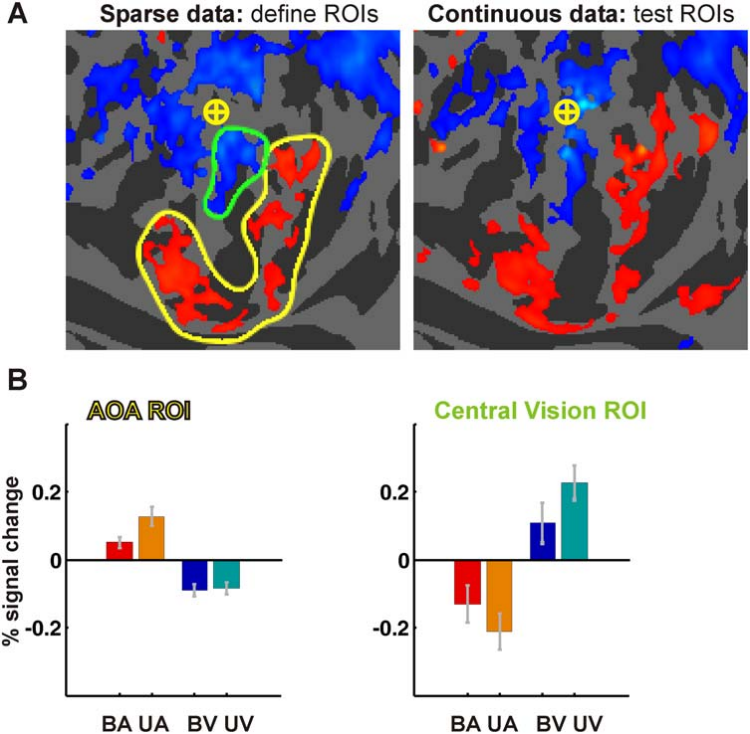
Region of interest (ROI) analyses. (A) Left: ARM activation maps from the sparse imaging data, plotted on the mean curvature map of the left hemisphere. The color scale and statistical thresholds are the same as in Figure 3. All significant voxels circumscribed by the yellow and green lines were designated as the AOA and central vision ROIs, respectively. Right: activation map from the continuous imaging data set used to analyze the ROIs, illustrated using identical thresholds. (B) Mean percent signal change for the four main task conditions in continuous imaging sessions: bimodal auditory (BA), unimodal auditory (UA), bimodal visual (BV) and unimodal visual (UV). A significant BA-BV difference indicates an ARM; a significant BV-UV difference indicates an auditory SDA; a BA-BV difference represents a visual SDA. The AOA ROI response was greatest when subjects attended to sounds in the absence of visual stimuli (UA condition), and showed no auditory SDA. Bars show standard errors of the mean.

he mean responses from the two ROIs (AOA and central vision ARM) during the four task conditions (BA bimodal stimulation, auditory attention condition, UA unimodal auditory, BV bimodal visual, UV unimodal visual) are plotted in Figure 6. Figure 6A shows the left hemisphere ARM activation map from the sparse imaging data, in which the ROIs are composed of all activated pixels falling within the outlined regions. The corresponding map from the continuous imaging data (used to analyze the ROIs) is shown alongside. The average responses from both ROIs during the four task conditions (UA, BA, UV and BV) are plotted in Figure 6B. In these plots responses were averaged across corresponding (but independently defined) ROIs from both hemispheres.

The AOA ROI did not respond to the presence of unattended sounds. Activations in the AOA ROI did not differ in UV and BV conditions, (F_(1,8)_ = 0.42, p = 0.54) showing that unattended auditory stimuli did not result in significant AOA generation. Moreover, activations in the AOA ROI were not affected by the intensity (F_(1,8)_ = 1.70, p>0.2), spatial location (F_(1,8)_ = 0.05, p>0.9) or frequency (F_(2,16)_ = 3.44, p>0.05) of unattended sounds.

In contrast, activations in the AOA ROI were significantly enhanced during attention to the auditory modality (BA vs. BV, F_(1,8)_ = 21.34, p<0.003). A comparison of the two auditory task conditions (UA and BA) revealed larger AOAs during the unimodal auditory attention condition when *no* visual stimuli were present (F_(1,8)_ = 8.86, p<0.02) suggesting that unattended visual stimuli inhibited AOA responses. The AOA ROI was not sensitive to the type of visual stimulus: neither the bimodal conditions ANOVA (F_(1,8)_ = 2.30, p = 0.17) nor the visual task conditions ANOVA (F_(1,8)_ = 2.06, p = 0.18) showed main effects of visual stimulus type.

The only stimulus parameter that reliably modulated AOA ROI activity was sound intensity: right hemisphere AOAs were larger during the more difficult auditory tasks with low-intensity sounds (F_(1,8)_ = 14.60, p<0.01). In the two bimodal conditions low-intensity sounds also evoked greater AOAs than high-intensity sounds (F_(1,8)_ = 8.73, p<0.02), with a similar right-hemisphere bias (F_(1,8)_ = 6.73, p<0.05).

### Relationship of AOAs to task switching at the beginning and end of stimulus blocks

**Figure 7:**
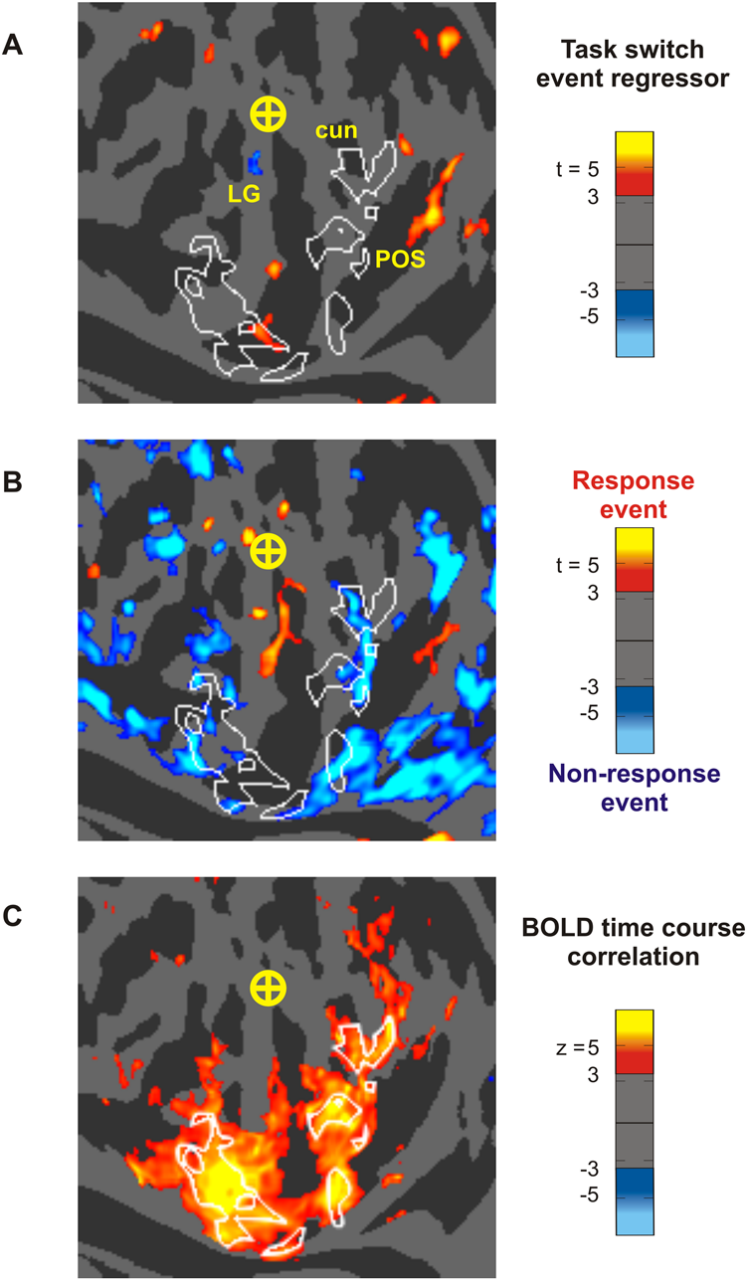
Task-related processes and auditory occipital activations. (A) *Task-switching*. Event-related time course regressors modeled activations associated with block termination and switching between auditory and visual tasks. Shown is the left hemisphere map from the continuous imaging data. Significant AOA regions (white outlines) overlapped very little with regions activated by task switching (red voxels). (B) *Auditory target detection*. Event-related time course regressors modeled button presses to targets during auditory attention blocks (red/yellow) as well as the intervals during which no responses were made (blue/cyan). Left hemisphere map is shown. AOA regions were not activated by target detection. (C) *Inhibition by foveal visual cortex*. Mixed-effects z-scores for the average correlation coefficient between the time course of each surface voxel and the mean time course of the AOA ROI, during unimodal visual conditions. Note the absence of significant correlations with central visual field voxels (region surrounding the circled cross). Left hemisphere map is shown.

Figure 7A shows the task switching regressor contrast map for the left hemisphere. There was no evidence of AOAs being associated with attentional transitions at the beginning or end of stimulation blocks.

### Relationship of AOAs to sustained auditory attention versus target detection responses

Figure 7B shows that target detection produced little activation within the AOA ROI. Thus, AOAs appeared to primarily reflect tonic attention-related activity rather then activity specifically related to target detection.

### Functional connectivity of AOA ROIs

The results of a partial correlation analysis using the mean AOA ROI as the seed are shown in Figure 7C. AOAs showed no significant correlations with activity in foveal visual cortex. However, the second partial correlation analysis showed a significant positive correlation (r = 0.08; t_8_ = 3.34, p<0.02) between activation in the AOA ROI and auditory cortex. This supports the hypothesis that AOAs are components of a network of brain regions engaged when subjects actively listen to sounds.

## Discussion

### Cognitive factors contributing to AOAs

In this study, AOAs depended critically on the engagement of auditory attention. AOAs were not generated by unattended sounds during visual attention conditions, regardless of sound intensity, location or frequency. In contrast, reliable AOAs were found in all subjects when they actively discriminated sounds. AOA magnitudes were not influenced by sound frequency or location, suggesting that they did not reflect the analysis of acoustic features.

The only acoustic parameter that modulated AOA magnitudes did so in a manner more consistent with an attentional account of AOA function than with a sensory role. AOAs were larger in blocks with low intensity sounds than in blocks with high intensity sounds. This effect is the opposite of fMRI sound intensity effects that are observed in core auditory sensory regions [74]–[79]. Sound intensity was also the only acoustic parameter that affected behavioral performance. Thus, one explanation of AOA enhancements to low-intensity sounds is that they reflected the increased engagement of sustained auditory attention during the more difficult low-intensity task conditions.

AOAs were localized to regions of visual cortex with visual receptive fields sensitive to stimuli in the far periphery [66], [80]–[82]. Lesions of these regions impair sound localization performance [35], and transient disruptions in processing in these regions from transcranial magnetic stimulation impairs performance on sound localization tasks [36]. The fact that AOA magnitudes were greater during behaviorally difficult blocks with low sound intensity suggests that AOAs are associated with auditory performance in sighted subjects, as has previously been reported in the blind [4], [6], [8], [19], [20]. The current results show that reliable AOAs can occur during non-spatial auditory discrimination tasks in sighted subjects, consistent with incidental reports of AOAs in previous studies of non-spatial attention tasks [40], [43], [83], [84].

One common feature of experiments in which AOAs are detected in sighted subjects is that sounds were delivered through earphones. In contrast, decreased occipital activations have been reported during auditory attention tasks when sounds were presented through visible loudspeakers located in the frontal spatial plane [6], [8]. These results suggest that when attention is directed to sound sources that are subjectively localized outside the visual field (as when sounds are delivered through headphones) peripheral regions of visual cortex are activated. Thus, AOAs may represent a special case of location-specific activation of visual cortex associated with cross-modal attention to spatial locations outside the visual field [85], [86]. As in previous reports, we found no consistent difference in the distribution of AOAs over the two hemispheres when sounds were delivered to one ear or the other [37]. This lack of spatial specificity suggests that invisible sound sources may prime peripheral visual cortex bilaterally, perhaps because stimuli localized outside the visual field can enter the visual field from unpredictable directions.

### AOAs in blind and sighted subjects

This study adds to growing evidence that AOAs occur in sighted as well as in blind subjects. It is now well-established that blind individuals, especially the congenitally or early blind, often have superior auditory task performance and larger AOAs than those found in sighted subjects [19]. The enhanced auditory performance of blind individuals is especially pronounced for sounds presented in the peripheral auditory field [24], [25]. Conversely, deaf individuals exhibit enhanced visual target detection, but only in the visual periphery [87], [88].

Enhanced performance in the blind may reflect cortical reorganization consequent to the disruption of normal visual input to the occipital lobe [30]. Recent studies [4] have suggested that AOAs in the blind may be mediated by anatomical projections between auditory association cortex and retinotopic visual cortex [11]. These projections terminate preferentially in peripheral visual cortex [10], [89], [90] and may play a role in the functional coupling of auditory and visual processing [12] seen in the current experiment. Enhanced development or utilization of these pathways may explain why blind individuals outperform sighted subjects in sound-localization tasks, but only when sounds are presented in peripheral locations [24].

### The relationship of AOAs to visual and auditory attention

Auditory signals can deactivate central regions of visual cortex that are activated by foveally presented visual stimuli [91], [92]. These deactivations depend on auditory attention [93]–[95] and are enhanced in conditions with greater auditory attentional load [96]. Since we generated AOAs using comparisons of visual versus auditory attention conditions, AOAs may have reflected the release from the inhibition of the peripheral visual cortex that has been hypothesized to occur when subjects attend to foveally presented stimuli [70], [71], [97]. This explanation is consistent with the observation that unattended visual stimuli reduced AOAs. Unattended visual stimuli would activate central visual cortex and simultaneously inhibit activations in peripheral visual regions.

However, the inhibition hypothesis predicts that there should be a systematic negative correlation between the magnitude of foveal visual cortex activations and the magnitude of AOAs. We found no significant correlations between AOAs and activations in the central vision ROI, suggesting that AOAs are not a direct consequence of inhibition exerted by foveal visual cortex. Rather, AOAs showed significant functional coupling with attention-related activations in auditory cortex.

Jack and colleagues [56] mapped AOAs to the cortical surface during tone discrimination tasks and found activation in retinotopic peripheral visual cortex, as in the current study. They also found that similar AOAs were produced following attended auditory response cues during visual discrimination tasks and when subjects produced self-generated responses in the absence of any auditory stimulation (i.e., after silently counting). It was proposed that these activations reflected top-down modulations of visual cortex associated with task completion at block transitions [98]–[100]. However, in the current study, we found no evidence of AOAs at block transitions, nor were AOAs associated with responses to auditory task targets. Thus, an alternative explanation of Jack et al's findings is that the AOAs observed reflected auditory attention to task-relevant auditory cues and the activation of the auditory attention network during silent counting [101].

Finally, we should note that the relationship between AOAs and auditory performance does not imply that occipital cortex need always be engaged by auditory attention. The efferent projections from auditory cortex to V1 in the macaque suggest that AOAs reflect the downstream modulation of peripheral visual cortex consequent to attention-related modulations in auditory cortex, of the sort observed in the current experiment (see Fig. 4) [79].

### Conclusions

Auditory occipital activations (AOAs) were found to depend strictly on auditory attention, and were not elicited by unattended sounds regardless of their acoustic properties. AOAs occurred reliably in auditory attention conditions and were enhanced during attention to unimodal auditory sequences and during the more difficult auditory-attention conditions with low-intensity sounds. AOAs were unrelated to activations in central visual cortex but showed significant functional coupling with attention-related activations in auditory cortex. Our results suggest that visual cortex subserving the far periphery is consistently engaged when subjects attended to sound sources outside the field of view. Crossmodal interactions between sensory cortices may indeed be the rule and not the exception in perception [102], and focusing on the attentional demands of perceptual tasks in neuroimaging studies may reveal increasing evidence of such effects.

## Supporting Information

Figure S1 Partial correlation analysis of auditory occipital activations and auditory cortex ROIs. The partial correlation under all task conditions was computed for the AOA ROI (all activated voxels within yellow outline) and an auditory cortex ROI (solid yellow region) located in Heschl's gyrus (HG) and the superior temporal gyrus (STG). The auditory cortex ROI was defined, using the data from sparse image acquisitions sessions, by subtracting responses during unimodal visual (UV) blocks from bimodal visual (BV) blocks. This ROI included all voxels meeting the three criteria of z>5.88 (p≪0.001), percent signal change >0.1% and cluster size 200 cortical surface voxels, and represented the auditory cortex region responding most strongly to unattended sounds. Data from the continuous image acquisition sessions were used to calculate the correlation while partialling out the global signal (means of both entire hemispheres) and head motion parameters; signal from an ROI defined as all visual ARM voxels in the posterior occipital region (all activated voxels within green outline); and indicator variables for bimodal vs. unimodal blocks and for auditory vs. visual blocks. The activation map shows the auditory (red) and visual (blue) ARM contrast using sparse image acquisition data from the left hemisphere; it is identical to the map in Figure 4. TP temporal pole, FG fusiform gyrus, IPS intraparietal sulcus, CC corpus callosum, CentS central sulcus. A circled cross indicates the occipital pole.
(0.92 MB TIF)
