# Supplementary figures and images for: Auditory Attention Activates Peripheral Visual Cortex
Source: PLoS One. 2009 Feb 27;4(2):e4645. doi: 10.1371/journal.pone.0004645 (PMC2644787; doi:10.1371/journal.pone.0004645)

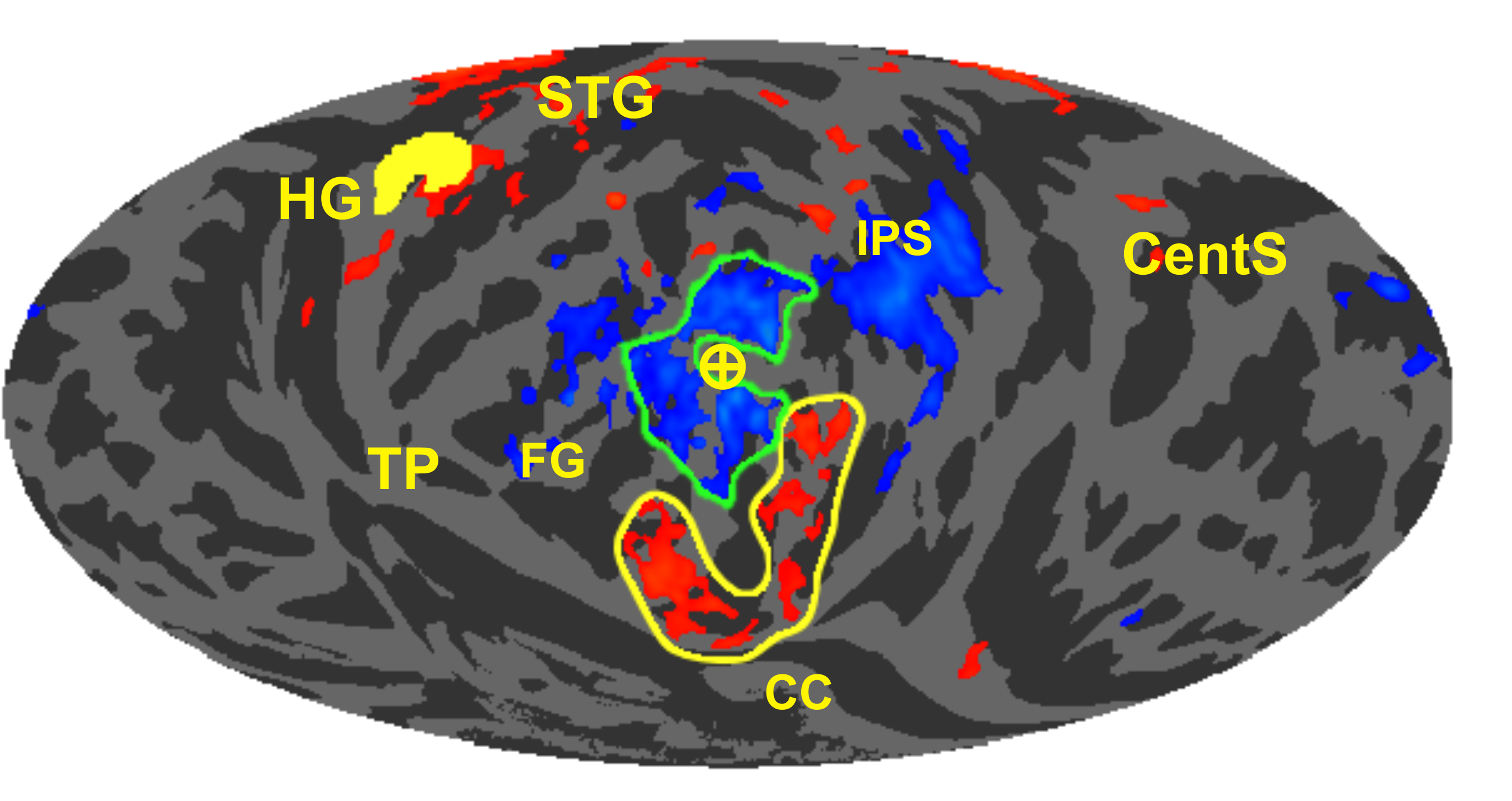

Supplement: Figure S1 — Partial correlation analysis of auditory occipital activations and auditory cortex ROIs. The partial correlation under all task conditions was computed for the AOA ROI (all activated voxels within yellow outline) and an auditory cortex ROI (solid yellow region) located in Heschl's gyrus (HG) and the superior temporal gyrus (STG). The auditory cortex ROI was defined, using the data from sparse image acquisitions sessions, by subtracting responses during unimodal visual (UV) blocks from bimodal visual (BV) blocks. This ROI included all voxels meeting the three criteria of z>5.88 (p≪0.001), percent signal change >0.1% and cluster size 200 cortical surface voxels, and represented the auditory cortex region responding most strongly to unattended sounds. Data from the continuous image acquisition sessions were used to calculate the correlation while partialling out the global signal (means of both entire hemispheres) and head motion parameters; signal from an ROI defined as all visual ARM voxels in the posterior occipital region (all activated voxels within green outline); and indicator variables for bimodal vs. unimodal blocks and for auditory vs. visual blocks. The activation map shows the auditory (red) and visual (blue) ARM contrast using sparse image acquisition data from the left hemisphere; it is identical to the map in Figure 4. TP temporal pole, FG fusiform gyrus, IPS intraparietal sulcus, CC corpus callosum, CentS central sulcus. A circled cross indicates the occipital pole. (0.92 MB TIF) [file pone.0004645.s001.tif]
